# Bioavailability of Dietary Omega-3 Fatty Acids Added to a Variety of Sausages in Healthy Individuals

**DOI:** 10.3390/nu9060629

**Published:** 2017-06-19

**Authors:** Anton Köhler, Johanna Heinrich, Clemens von Schacky

**Affiliations:** Department of Preventive Cardiology, Medizinische Klinik and Poliklinik I, Campus Innenstadt, Ludwig Maximilians University, Ziemssen str.1, D-80336 Munich, Germany; anton.koehler@med.uni-muenchen.de (A.K.); heinrichjo@gmx.de (J.H.)

**Keywords:** omega-3 index, omega-3 fatty acids, eicosapentaenoic acid, docosahexaenoic acid, alpha-linolenic acid

## Abstract

A low Omega-3 Index (eicosapentaenoic acid (EPA) and docosahexaenoic acid (DHA) in erythrocytes) is associated with cardiac, cerebral, and other health issues. Intake of EPA and DHA, but not of alpha-linolenic acid (ALA), increases the Omega-3 Index. We investigated bioavailability, safety, palatability and tolerability of EPA and DHA in a novel source: a variety of sausages. We screened 96 healthy volunteers, and recruited 44 with an Omega-3 Index <5%. Participants were randomly assigned to receive a variety of sausages enriched with approximately 250 mg EPA and DHA per 80 g (*n* = 22) daily for 8 weeks, or matching placebo sausages (*n* = 22). All sausages contained approximately 250 mg ALA/80 g. In the verum group, the mean Omega-3 Index increased from 4.18 ± 0.54 to 5.72 ± 0.66% (*p* < 0.001), while it remained unchanged in the placebo group. While ALA levels increased only in the placebo group, DPA levels increased in both groups. Inter-individual variability in the response was large. The mean increase of the Omega-3 Index per intake of EPA and DHA we observed was higher than for other sources previously studied, indicating superior bioavailability. As increasing production of EPA and DHA is difficult, improvements of bioavailability can facilitate reaching the target range for the Omega-3 Index (8–11%).

## 1. Introduction

The two omega-3 fatty acids eicosapentaenoic acid (EPA, 20:5*n*-3) and docosahexaenoic acid (DHA, 22:6*n*-3) fulfill important structural and functional roles in cell membranes [1,2]. Tissue levels of, and thus the status of an individual in, EPA and DHA are represented by the Omega-3 Index, the sum of EPA and DHA in erythrocytes, as analyzed with a highly standardized method [3]. A low Omega-3 Index is associated with elevated total and cardiovascular mortality [3,4], major depression [5,6], impairments of cognitive function [7,8], and other health issues.

Theoretically, increasing intake of the plant-derived omega-3 fatty acid alpha-linolenic acid (ALA) increases levels of EPA and DHA. However, conversion of ALA to EPA is poor, and ALA does not increase DHA [9].

Increasing intake of EPA and DHA is the most effective way to increase the Omega-3 Index. However, traditional sources of EPA and DHA have disappeared or are disappearing from our diet. An example is the disappearance of DHA-containing cattle brain, due to the threat of bovine spongiform encephalitis. Another is the reduction in the EPA and DHA content of farmed fish, due their replacement by other fatty acids in fish farming. Therefore, novel food sources of EPA and DHA need to be developed. One way is feeding EPA and DHA to animals to enrich their meat with both fatty acids; another is by feeding alpha-linolenic acid to animals capable of converting alpha-linolenic acid to EPA and DHA [10,11,12,13,14,15,16,17,18]. Clearly, these novel food products not only need to contain EPA and DHA, but EPA and DHA must also be bioavailable. Bioavailability is the extent to which a nutrient can be absorbed and transported into systemic circulation or site of physiological activity. Bioavailability of EPA and DHA varies with a number of factors, like specific emulsification, inter-individual differences, concomitant food intake or chemical form [19]. This makes it imperative to demonstrate bioavailability of EPA and DHA in any new food source. Moreover, the safety, tolerability and palatability of any new food source needs to be documented. 

The aim of the present study was to investigate how daily intake of a variety of sausages supplemented with EPA and DHA (approximately 250 mg EPA and DHA and 240 mg ALA per day) influences the Omega-3 Index, as compared to matching sausages supplemented only with ALA (approximately 257 mg per day). Safety, tolerability, and palatability of the sausages were also assessed. Finally, in a review of the pertinent literature, and using the change in Omega-3 Index per amount of EPA and DHA ingested as a parameter, we compared the bioavailability we found, to the bioavailability of other products.

## 2. Participants and Methods

### 2.1. Study Participants

From June until October 2014, healthy adults were asked to participate. Inclusion criteria were (1) age (20–60 years); (2) willingness to eat 80 g/day of the sausage; (3) a low Omega-3 Index (<5%); (4) adequate fluency in German or English to complete baseline and follow-up interviews; (5) stable intake of food containing EPA and DHA before and during the study. Exclusion criteria were (1) regular intake of omega-3 fatty acids supplements or >2 portions of fatty fish per week; (2) serious bleeding disorder; (3) any acute and life-threatening condition; (4) significant medical co-morbidity; (5) seriously limited life expectancy; (6) insulin-treated diabetes mellitus; (7) Body-Mass-Index (BMI) > 30 kg/m^2^; (8) allergy, intolerance or history of hypersensitivity to components of study intervention; (9) pregnancy, breast-feeding or childbearing potential without medically accepted method of contraception; (10) use of any investigational agent within 30 days prior to screening visit; (11) known drug- or alcohol abuse/dependence within the past 2 years. Only participants willing to adhere to all aspects of the study protocol were recruited.

The study protocol was approved by the Ethics Committee of the Faculty of Medicine of the Ludwig Maximilians-University of Munich and registered on Clinicaltrials.gov (NCT02148835). All procedures were in accordance with current ethical standards of the Helsinki Declaration, and the study was conducted according to Good Clinical Practice. Written informed consent was obtained from all individuals before participating, allowing analysis of all clinical and laboratory data mentioned in the present paper. The study was initiated, designed, conducted and evaluated by the investigators, and the sponsor had no role in study design, data acquisition or evaluation, or in preparation of the manuscript.

### 2.2. Study Design

The present study was a randomised, double-blind, placebo-controlled, mono-center comparison of two matching groups of sausages (verum vs. placebo, Table 1). Randomisation was computer-generated. Primary endpoint was a change in the Omega-3 Index. Secondary endpoints were safety, tolerability, and palatability of the sausages, as assessed by a questionnaire including visual analogue scale (1 stands for very poor and 10 for very good), and changes in blood lipids (cholesterol, high-density lipoprotein cholesterol (HDL) and low-density lipoprotein cholesterol (LDL), total cholesterol, triglyceride), blood glucose, HbA1c, liver enzymes (aspartate aminotransferase, alanine aminotransferase and g-glutamyl transferase), creatinine, heart rate, blood pressure, BMI and WHR (waist to hip ratio) and changes in the fatty acid composition of erythrocytes.

After screening (t0) an 8-week intervention period followed. At run-in (t1) and at end-of-study visit at week 8 (t8), blood samples and clinical parameters were obtained. Furthermore, participants were asked about their food habits, especially about their fish intake or intake of omega-3 fatty acids. Venous blood was collected by venipuncture at t0 in a non-fasting state to measure the Omega-3 Index, and at t1 and t8 after an overnight fast to measure predefined biochemical parameters as shown in Table 2. At week 4 (t4), a telephone follow-up was conducted regarding palatability, adverse events, compliance and changes of food habits. Study participants were requested to ingest 80 g/day of sausages during the day at a time of their convenience and not to alter their current diet. Compliance was assessed by interrogation. 

The investigational product used was manufactured by Südbayerische Fleischwaren in Ingolstadt, Germany. Outer packing of verum and placebo products looked identical. Participants were given a variety of the products listed in Table 1. Participants were given 80 g packs for each day, which contained 500 kJ (120 kcal), 12 g protein, 0.8 g carbohydrates, 9 g total fat, of which 2.8 g were saturated fatty acids, 4.5 g monounsaturated fatty acids, 1.4 g polyunsaturated fatty acids and 0.66 g sodium (data supplied by the producer). All participants picked up a two-week supply of the experimental sausages at the study center every two weeks in a refrigerated container. Care was taken that all participants received an identical selection and amount of each of the sausages.

### 2.3. Laboratory Methods

Erythrocyte fatty acid composition was analysed according to the HS-Omega-3 Index^®^ methodology as previously described [20]. Other blood parameters were determined by the Department of Clinical Chemistry—Klinikum Innenstadt (Ludwig-Maximilians-University, Munich, Germany) using routine clinical chemistry methods.

### 2.4. Statistical Analyses

The power calculation was based on Köhler et al. [20], where 0.5 g EPA + DHA in the form of a triglyceride in a convenience drink was given for 8 weeks, and the Omega-3 Index rose from 4.37 ± 0.51% to 6.8 ± 1.45%. Due to the lower dose (250 mg/day) and the form of an ethyl-ester (at the time thought to be approximately 50% less bioavailable [21]), a fourth of the effect size within 8 weeks was anticipated, i.e., a change of +0.61%. The usual assumptions were made (alpha = 5%, power = 80%). According to a web-based case estimate for this parallel-design study [22], the necessary sample size was 44. The probability was 81% that the study would detect a treatment difference at a one-sided 0.05 significance level, if the true difference between treatments was 0.580 units; standard deviation of the response variable was assumed as 0.75. Analysis was by intention to treat. 

Results are presented as means and standard deviations. Statistical differences were calculated using an unpaired *t*-test for comparison of intervention versus control, and a paired *t*-test for the comparison of baseline with end of the trial. Statistical differences in palatability and tolerability were calculated using the Mann–Whitney U test to analyse variables of ordinal scale. Differences with *p* values < 0.05 were considered statistically significant. Data were examined by IBM SPSS Statistics for windows (release 18.0, IBM, Chicago, IL, USA).

### 2.5. Review of the Literature

A list of publications based on the HS-Omega-3 Index^®^ is being continuously updated by one of us (C.v.S.). This list, currently containing 219 entries, was searched for intervention trials, and 32 were found. Only trials with a continuous substitution of EPA and DHA, documented chemical forms and dosages were selected. These trials were sorted according to the chemical form of EPA and DHA used in the trial. Additional criteria excerpted were baseline Omega-3 Index in %, change in Omega-3 Index in %, dose EPA + DHA given in mg/day, trial duration, and number of participants. Change in Omega-3 Index in % per 100 mg EPA + DHA given per day in the respective trial was calculated without correcting for trial duration. In a second step, the change in Omega-3 Index in % per 100 mg EPA + DHA given per day was related to trial duration and other factors possibly affecting bioavailability, like chemical form, matrix used (e.g., liquid emulsion, fish oil, fish meal, krill oil, etc.).

## 3. Results

### 3.1. Screened Group

**Figure 1:**
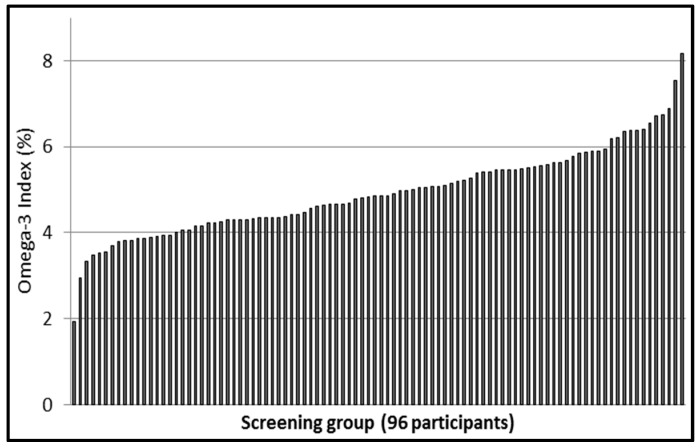
Omega-3 Indices in ascending order of 96 screened participants.

A total of 96 participants were screened; 44 were randomized. Main exclusion criterion (about 83%) was an Omega-3 Index > 5.0%. Most of the individuals screened were university students, 60 male and 36 female, mean age 27.3 ± 7.2 years (female 28.6 ± 9.0 years, male 26.5 ± 5.8 years, *p* = 0.169) and mean Omega-3 Index 4.93 ± 1.00% (female 5.13 ± 1.11%; male 4.80 ± 0.92%, *p* = 0.119). The distribution of the Omega-3 Index among the screened participants is shown in Figure 1.

### 3.2. Study Group

**Figure 2:**
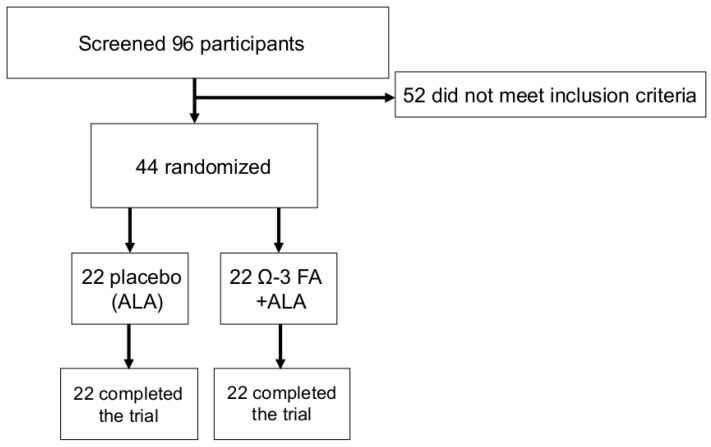
Study flow diagram.

Study design and flow of participants are shown in Figure 2. A total of 22 participants, mean age 26.4 ± 8.0 years (9 female, 28.7 ± 12.1 years and 13 male, 24.9 ± 2.9 years, *p* = 0.283), were randomized to the verum group and received verum sausages (Table 1). One participant in the verum group had the diagnosis of a heterozygote Factor V Leiden mutation (without any thromboembolic events in the past), two had mild hypertension (one treated with beta-blocker and one with ACE-inhibitor), three participants reported different forms of pollen allergy, one a lactose intolerance and one an intolerance against hen’s egg ovalbumin.

A total of 22 participants, mean age 25.6 ± 4.1 years (6 female, 28.0 ± 4.0 years and 16 male, 24.8 ± 3.8 years, *p* = 0.094), were randomized to the placebo group and received placebo sausages (Table 1). One participant in the placebo group was diagnosed with mild depression (treated with low dose Escitalopram) and hypothyreosis (treated with L-Thyroxin), four participants reported different forms of pollen allergy, one an insect allergy and two, lactose intolerance.

At baseline, there were no differences in clinical and biochemical parameters between the two groups (Table 2).

### 3.3. Primary End Point

**Figure 3:**
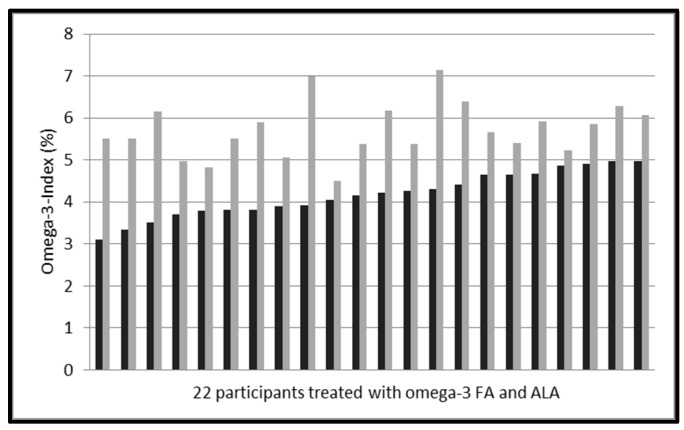
Omega-3 Indices of 22 healthy participants pre and post treatment with EPA and DHA (approximately 250 mg/day) as ethyl-esters and ALA (240 mg/day) for 8 weeks (black: pre treatment, grey: post treatment). Mean values pre treatment 4.18 ± 0.54% and post treatment 5.72 ± 0.66% (*p* ≤ 0.001).

In the verum group, the daily intake of approximately 250 mg EPA and DHA as ethyl-esters in a variety of sausages for 8 weeks increased the mean Omega-3 Index from 4.18 ± 0.54% to 5.72 ± 0.66% (*p* < 0.001, Table 3, Figure 3). Mean increase was 1.54 ± 0.75%, with a large inter-individual variability (range 0.36–3.07%). The increase was independent of BMI (*r* = −0.09, *p* = 0.699) and age (*r* = −0.40, *p* = 0.063), and there was no difference in response between female and male individuals (*p* = 0.637).

**Figure 4:**
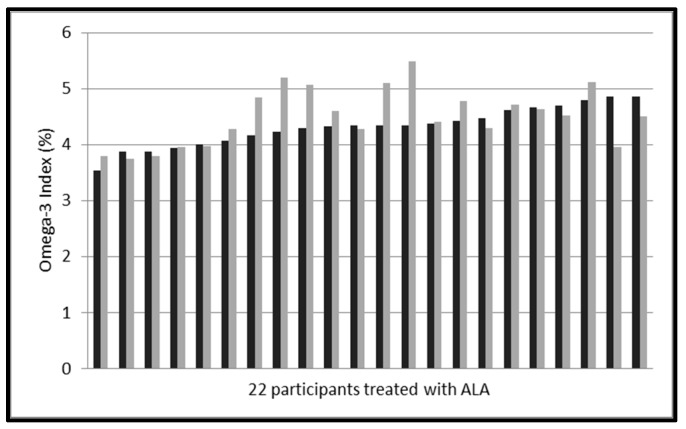
Omega-3 Indices of 22 participants pre and post treatment with ALA (257 mg/day for 8 weeks (black: pre treatment, grey: post treatment). Mean values pre treatment 4.32 ± 0.35% and post treatment 4.50 ± 0.51% (*p* = 0.089).

In the placebo group the Omega-3 Index did not change after 8 weeks intervention (4.32 ± 0.35% to 4.50 ± 0.51%, *p* = 0.089) (Table 3, Figure 4).

### 3.4. Secondary Endpoints, Adverse Events and Laboratory Safety Test

**Figure 5:**
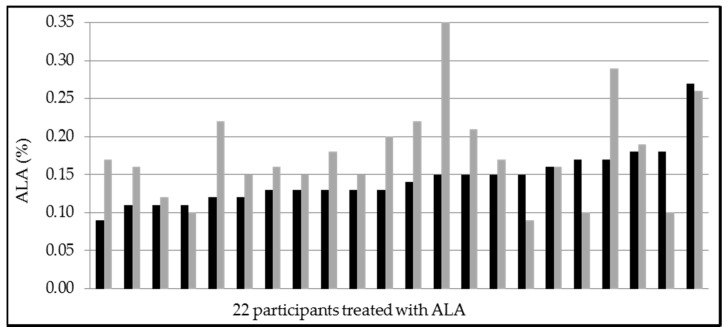
Alpha-linolenic acid-levels of 22 participants pre and post treatment with ALA (257 mg/day) for 8 weeks (black: pre treatment, grey: post treatment). Mean values pre treatment 0.14 ± 0.04% and post treatment 0.18 ± 0.08% (*p* = 0.038).

**Figure 6:**
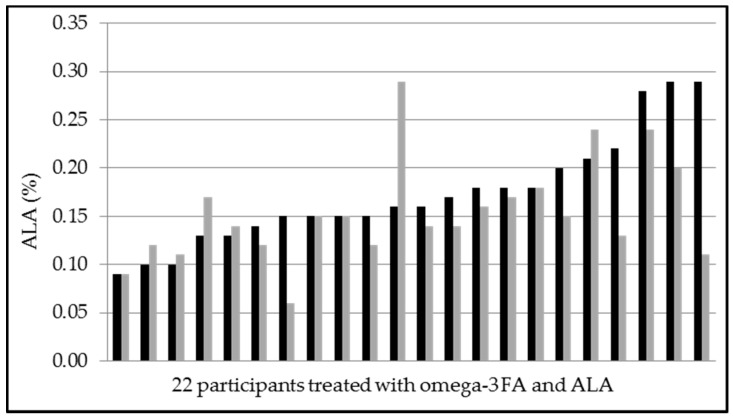
Alpha-linolenic acid-levels of 22 participants pre and post treatment with EPA and DHA (approximately 250 mg/day) and ALA (240 mg/day) for 8 weeks (black: pre treatment, grey: post treatment). Mean values pre treatment 0.17 ± 0.06% and post treatment 0.15 ± 0.05% (*p* = 0.141).

After 8 weeks of intervention, the fatty acid composition in erythrocytes showed significant changes in verum vs. placebo (Table 3). Both EPA and DHA increased in the verum group. Docosapentaenoic acid (DPA)-levels increased in both groups. ALA increased only in the placebo group (mean increase 0.04 ± 0.08%, *p* = 0.038) (Figure 5), whereas in the verum group ALA levels decreased (mean decrease −0.02 ± 0.06%, *p* = 0.141) (Figure 6).

At telephone follow-up at week 4 (t4) in the verum group, one participant was diagnosed with an influenza infection and three had mild symptoms of a cold. In the placebo group, one had mild gastrointestinal symptoms with sickness and one had a viral infection with fever, diarrhea and emesis. No participant received any antiviral or antibiotic treatment. All documented adverse events were mild and estimated as not related to the investigational product. In the verum group nine participants (placebo group five participants) reported that “Weißwurst” and one participant that “Bratwurst” (one in placebo group) and “Leberkäse” were of no good taste. Palatability (verum 7.0 (4.0; 10.0), placebo 7.0 (3.0; 10.0); *p* = 0.922) and tolerability (verum 10.0 (7.0; 10.0), placebo 10.0 (7.0; 10.0); *p* = 0.870), both assessed by a visual analog scale, were not different between the two groups.

At end-of-study visit (t8) one individual of the placebo group reported mild digestive discomfort. This adverse event was estimated as possibly related to the investigational product. Two individuals reported mild symptoms of a cold. From the verum group, no adverse events were reported at t8. Palatability (verum 7.0 (4.0; 9.0), placebo 7.0 (3.0; 10.0), *p* = 0.465) and tolerability (verum 10.0 (6.0; 10.0), placebo 10.0 (7.0; 10.0); *p* = 0.816) again did not differ between the two groups.

As shown in Table 2, none of the measured clinical and biochemical parameters changed between the placebo and the verum groups after 8 weeks intervention.

In both groups no individual altered the intake of fatty fish, the current diet or concomitant medication during the study period. Compliance, assessed by interrogation, was very good in both groups. All 44 participants finished the trial and no serious adverse events were observed.

### 3.5. Review of the Literature

**Figure 7:**
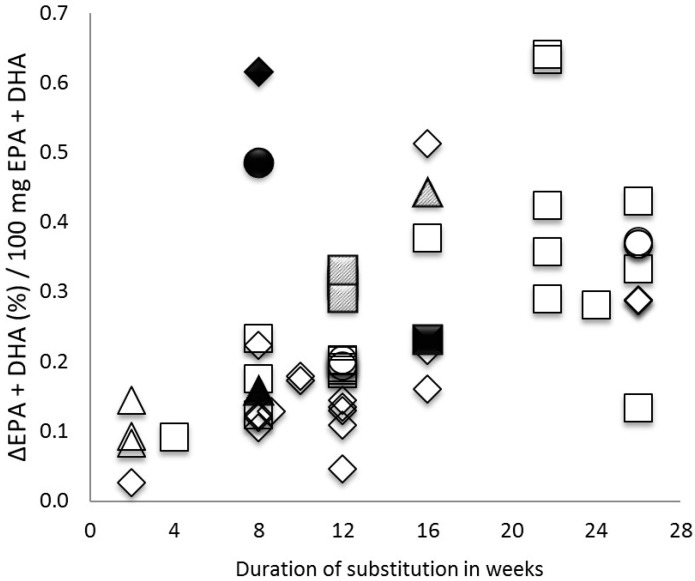
Comparison of bioavailability of EPA + DHA given in different chemical forms and for different durations. This figure shows the increase of the Omega-3 Index (∆EPA + DHA (%)) per 100 mg EPA and DHA substituted in trials with different duration and use of different chemical binding forms. Abbreviations: black triangle = acylglycerol; white triangle = liquid emulsion formulation; shaded triangle = fish meal; black square = microalgae oil; white square = fish oil, shaded square = krill oil; black circle = triacylglycerides; white circle = re-esterfied triacylglycerides; white diamond = ethyl esters; black diamond = triOMEG-trial. References as in Table 4.

As detailed in Table 4, in trials of at least 8 weeks duration, the increase in Omega-3 Index per 100 mg EPA + DHA given per day varied from 0.11 to 0.61 (this trial), with no apparent relation to chemical form of EPA + DHA. The increase in Omega-3 Index per 100 mg EPA + DHA given for 8 weeks in the sausages studied here was comparable to five months of a fish oil in capsules (Figure 7), illustrating the superior bioavailability of the novel source of EPA + DHA studied here. More details can be found in Figure 7.

## 4. Discussion

After daily intake of approximately 250 mg EPA and DHA as ethyl-esters in a variety of sausages for 8 weeks, the Omega-3 Index increased significantly. There was a large inter-individual variability in response, although the study population was recruited based on an Omega-3 Index < 5% (Figure 3). In terms of safety, tolerability and palatability no major problems were noted.

### 4.1. Screened Individuals

Of 96 individuals screened, only one had an Omega-3 Index in the target range of 8–11% [1,2]. In 106 German athletes of a similar age, we also found only one individual in the target range [55]. The mean Omega-3 Index in the screened individuals was 4.93 ± 1.00%, while it was 4.97 ± 1.19% in the athletes [55]. In both populations, as in every population so far studied, the Omega-3 Index had a Gaussian, statistically normal distribution (Figure 1) [55]. Most of the screened individuals in our study were university students and it has been reported that a higher education level is associated with a higher Omega-3 Index [56,57]. Therefore, in an unselected German population, an even lower mean Omega-3 Index might be found. Our finding is consistent with the low intake of fish in individuals up to age 30 in Germany [58]. The low mean Omega-3 Index puts the individuals screened at risk for cardiovascular events, issues of impaired complex brain function, and major depression [3,56].

### 4.2. Choice of Product

The choice of sausages as a vehicle for EPA and DHA may seem odd. However, in Germany, sausages are part of the everyday diet. Germany has about 1200 different sausages, and some historians consider eating sausages a defining element for being German [59]. Therefore, selecting sausages as a vehicle for EPA and DHA in Germany was a logical choice. In our trial, compliance with study procedures was not an issue. Our trial therefore demonstrates that it is possible to increase the amount of EPA and DHA in Germans’ everyday diet by using sausages. The increase in the Omega-3 Index in 8 weeks from mean 4.18 ± 0.54% to 5.72 ± 0.66%, although significant, was not sufficient to bring the study participants into the target range of 8–11% [3,56]. Therefore, we consider our approach one option to alleviate the widespread deficit in EPA and DHA, but no solution to it. Moreover, our approach may not be as viable in other countries or in other populations, like vegetarians.

### 4.3. Bioavailability of the Investigational Product

In the present study, a daily intake of approximately 250 mg EPA and DHA as ethyl-esters via sausages for 8 weeks increased the Omega-3 Index from mean 4.18 ± 0.54% to mean 5.72 ± 0.66% (*p* < 0.001). Compared to baseline levels, we detected a mean increase of the Omega-3 Index of 1.54 ± 0.75%. This was much higher than the expected 0.6%. Individuals with a low baseline Omega-3 Index and low body weight experience a greater increase of the Omega-3 Index as a result of substitution with EPA and DHA than individuals with a high baseline and/or a high body weight [3,56,60]. Therefore, the low baseline Omega-3 Index, and the fact that we only included normal weight adults, might partly explain, why we observed a larger response than expected. 

Differences in the lipid structure in which EPA and DHA are ingested may influence their bioavailability and accumulation within lipid pools. Bioavailability of *n*-3 fatty acids is thought to vary overall by a factor of two, depending on their chemical form: phospholipid > recombined triglyceride > triglyceride > free fatty acid > ethyl-ester [12,61,62,63,64,65]. The lower bioavailability of EPA and DHA from ethyl-esters than from triglycerides is in accord with the demonstration that pancreatic lipase hydrolyses ethyl-esters to a lesser degree than triglycerides and at a slower rate [63,64,66]. Recently, we questioned this thought based on the results of a single dose, randomized, double-blind cross-over trial comparing similar doses of krill oil, krill meal, and fish oil [67]. We found bioavailability of EPA and DHA in krill oil superior to their bioavailability in krill meal or fish oil, while the bioavailabilities of EPA and DHA in krill meal or fish oil were comparable. In the krill oil and krill meal studied, EPA and DHA were bound in phospholipids, while in the fish oil, EPA and DHA were bound in triglycerides. Therefore, other factors seem to impact bioavailability more than chemical form. Indeed, when properly emulsified, bioavailability of EPA and DHA ethyl-ester was up to 21-fold better than when ingested unemulsified in a capsule (as marketed as a drug) [23] Lately, West et al. [68] showed for healthy individuals that differences in the chemical form of EPA and DHA have no relevant influence on bioavailability.

The relatively good bioavailability of *n*-3 fatty acids supplemented as ethyl-esters in various sausages might be explained by the findings of Nordøy et al., who found an equally good absorption of *n*-3 fatty acids from ethyl-esters and triglycerides when given as part of a lipid-rich meal [65]. The absorption of EPA and DHA from ethyl-esters is increased substantially by co-ingestion with a high-fat meal by enhanced assimilation of ethyl-esters [69].

Most of EPA and DHA are derived from marine sources, and production cannot be increased easily. New sources of EPA and DHA are currently identified and developed, such as krill, algae, and genetically modified plants. We consider improving bioavailability of EPA and DHA as important as developing new sources.

### 4.4. Variability in Response 

As depicted in Figure 3, baseline Omega-3 Index was rather homogeneous in the verum group. The increase of the Omega-3 Index in response to 250 mg EPA and DHA varied from 0.36–3.07%, which, based on our findings, would be difficult to explain by issues of compliance. Earlier, we demonstrated variability of bioavailability of EPA and DHA as a triglyceride in a convenience drink by a factor of 13 inter-individually [20]. In the meantime, however, this has been confirmed by us, and others [47,67]. While predicting a mean dose-response of the Omega-3 Index to supplementation with EPA and DHA in a population using complicated statistical models is possible, such prediction is basically impossible for an individual [20,47,67]. Smoking and physical activity have been reported to correlate inversely with the Omega-3 Index, and other factors, like genes, also play a role [3,19,56]. We suggest that these factors might also impact the response of the Omega-3 Index within an eight week study duration. Larger studies are necessary to clarify what defines the individual response of the Omega-3 Index in quantitative terms. 

### 4.5. Changes in ALA

All sausages were enriched with ALA, approximately 240 mg in the verum group and 257 mg in the placebo group. The amount of supplemented omega-3 fatty acids in the investigational products were not the same in both groups (Table 1). However, ALA (but not EPA or DHA) increased in the placebo group (and not in the verum group) and showed a large interindividual variability in response (Figure 5 and Figure 6). DPA increased similarly in both groups (Table 3). Our results support previous findings that the conversion of ALA into EPA is poor, and into DHA is not of the same importance in humans. We cannot explain our novel finding that ALA did not increase, when ingested with EPA and DHA. Although our study is underpowered to investigate all the reported differences in erythrocyte fatty acid composition, these apparent interactions in omega-3 fatty acid metabolism suggest further research.

### 4.6. Clinical Impact

Based on previous epidemiological findings, the increase of the Omega-3 Index in the verum group should translate into a reduced risk for clinical events like total mortality, cardiovascular endpoints, major depression, cognitive impairments, and other untoward events [3,6,56,70,71]. It has been notoriously difficult to translate this reduction of risk into reduction of events in pertinent intervention trials. We suggest the large inter-individual variability in the response of the Omega-3 Index to increased intake of EPA and DHA as one explanation for the neutral outcome of those trials. As discussed in more detail elsewhere, we think that aiming for a target range of the Omega-3 Index (e.g., 8–11%) using an individualized dose of EPA and DHA will be a fruitful approach for intervention trials [72].

### 4.7. Safety, Tolerability, and Palatability

Placebo-controlled intervention studies showed that side effects of substitution with omega-3 fatty acids are at placebo level [3,56,70,71,72]. Only one adverse event was reported in our placebo group (mild digestive discomfort) that was considered as possibly related to the investigational product. No serious adverse event was reported. The present study was too small to detect rare side effects. In our small trial, the omega-3 preparation and dose used were safe, of very good tolerability and of good palatability.

### 4.8. Strengths and Limitations

Strengths of the study include: (1) a homogenous study population; (2) good palatability and tolerability of the investigational product; (3) standardized fatty acid analysis using the Omega-3 Index; (4) trial design and reporting conform current standards [73].

Limitations include that the study was (1) a single-centre study; (2) too small to detect rare side effects; (3) only healthy adults were included; (4) with only eight weeks, relatively short in duration; (5) the investigational products contained slightly different amounts of supplemented omega-3 fatty acids; and (6) the study was underpowered for further investigation of changes in erythrocyte fatty acid composition.

### 4.9. Review of the Literature

In our review of intervention trials with EPA + DHA, we compared only trials that used an identical analytical procedure for the criterion compared. This analytical procedure, the HS-Omega-3 Index, is probably currently the most widely used, and we suggest it to be used in future investigations in order to make future results comparable to present results, and to facilitate clinical use of fatty acid analyses. 

For the sausages we investigated, we found a mean ∆EPA + DHA in % per 100 mg EPA + DHA eaten of 0.62%/100 mg, superior to bioavailability of EPA and DHA in other preparations studied so far (Figure 7, Table 4). For comparison of the trials with different durations and dosages of EPA and DHA the mean increase of EPA and DHA levels from baseline per 100 mg EPA and DHA substitution (∆EPA + DHA (%)/100 mg EPA + DHA) was calculated. Clearly, this is not a perfect parameter for comparison, because levelling-off of the dose response at higher levels has been observed [47], as well as for other reasons. However, only a parameter like this can make trial results comparable. Notably, the lowest value of ∆EPA + DHA in % per 100 mg EPA + DHA we found was 0.03 and the highest 0.64. Even among the 10 trials with the same duration (8 weeks), values showed a range between 0.11–0.62%/100 mg (dosages used between 250–3630 mg) (Figure 7). As an explanation, the chemical form can be neglected, as discussed above, and can also be seen in Table 4 and Figure 7. We suggest that bioavailability of EPA + DHA depends to some degree on the matrix in which EPA and DHA are ingested, since EPA and DHA in foods or emulsions seemed to have a higher bioavailability than EPA and DHA in capsules, and unemulsified (Table 4). However, we cannot exclude many other mechanisms, including, although not likely, even aspects of catabolism of EPA and DHA. Moreover, our review of the literature has many limitations, like differences in trial duration and dose, differences in number, age, gender and comorbidities of participants, and others. However, in light of the fact that improving bioavailability is a way of improving the Omega-3 Index without increasing production of EPA and DHA, we suggest intensifying research on this topic.

## 5. Conclusions

The present trial demonstrated that daily intake of approximately 250 mg EPA and DHA as ethyl-esters in a variety of sausages for eight weeks increased the Omega-3 Index. The dose and preparation used were well-tolerated. Corrected for dose and compared with other trials, the investigated product was very effective in increasing the Omega-3 Index. Clearly, the product we studied may not be useful for all populations. A large variability in response to EPA and DHA was observed, which remains to be explained mechanistically. Our findings question the wisdom of recommending a fixed dose of EPA + DHA, and support individualizing dosing and improving bioavailability to reach the proposed target range for the Omega-3 Index of 8–11%.

## Figures and Tables

**Table 1:** Amount of eicosapentaenoic acid (EPA) and docosahexaenoic acid (DHA) in mg/80 g (as eaten daily) of the investigational product.

Name of Sausage	Amount of EPA + DHA (mg/80 g)
Bratwurst	408
Gourmet Trio	274
Leberkäse fein dick	296
Leberkäse fein dünn	296
Lyoner	280
Paprika Lyoner	280
Weißwurst	280
Wiener	296

The daily dosage of the investigational product of 80 g sausage was enriched with the amounts EPA and DHA mentioned in Table 1 and 240 mg alpha-linolenic acid (ALA). The matching placebo product contained approximately 257 mg ALA per 80 g sausage. EPA and DHA were in the chemical form of an ethyl-ester, and produced from Anchovis oil by KD Pharma, Bexbach, Germany. ALA was in the chemical form of a triglyceride, and produced from rapeseed oil by Scheid AG, Überherrn, Germany.

**Table 2:** Clinical and biochemical parameters at baseline and end of study (Mean values ± standard deviation).

Parameter	Placebo (*n* = 22 *) t1	Verum (*n* = 22 *) t1	*p*	Placebo (*n* = 22) t8	Verum (*n* = 22) t8	*p*
BMI (kg/m^2^)	23.47 ± 1.99	24.27 ± 2.71	0.269	23.60 ± 1.99	24.24 ± 2.76	0.379
WHR	0.78 ± 0.05	0.81 ± 0.09	0.120	0.77 ± 0.05	0.80 ± 0.08	0.127
HR (bpm)	67.0 ± 10.5	68.1 ± 11.6	0.745	70.3 ± 11.3	71.3 ± 11.7	0.770
BP Sys (mmHg)	127.1 ± 9.7	129.4 ± 13.3	0.516	130.3 ± 10.0	128.9 ± 14.9	0.728
BP Dia (mmHg)	75.9 ± 7.4	79.8 ± 10.0	0.143	76.2 ± 6.8	81.2 ± 10.5	0.068
Blood glucose (mg/dL)	86.4 ± 6.8	85.5 ± 4.9	0.646	84.0 ± 8.1	86.7 ± 10.6	0.345
HbA1c (%)	4.93 ± 0.21	4.92 ± 0.28	0.952	5.03 ± 0.25	5.04 ± 0.28	0.955
Total cholesterol (mg/dL)	162.6 ± 22.2	163.3 ± 33.4	0.941	165.3 ± 28.2	168.4 ± 37.2	0.754
HDL cholesterol (mg/dL)	60.7 ± 12.3	61.1 ± 9.9	0.904	59.7 ± 10.3	61.4 ± 11.6	0.623
LDL cholesterol (mg/dL)	90.6 ± 25.3	88.4 ± 30.0	0.787	91.7 ± 27.2	91.1 ± 32.3	0.944
Triglyceride (mg/dL)	72.3 ± 26.4	87.2 ± 35.4	0.122	87.0 ± 42.0	100.8 ± 55.8	0.360
Creatinine (mg/dL)	0.94 ± 0.12	0.94 ± 0.13	0.907	0.94 ± 0.11	0.93 ± 0.13	0.903
AST (U/L)	23.8 ± 5.5	29.3 ± 24.6	0.318	21.8 ± 4.5	23.2 ± 6.2	0.393
ALT (U/L)	25.1 ± 9.6	24.7 ± 9.8	0.889	22.5 ± 7.9	24.2 ± 11.1	0.544
GGT (U/L)	20.2 ± 10.1	28.3 ± 26.5	0.198	20.9 ± 10.8	26.5 ± 18.5	0.232
Omega-3 Index (%)	4.32 ± 0.35	4.18 ± 0.54	0.318	4.50 ± 0.51	5.72 ± 0.66	<0.001

BMI, body-mass-index; WHR, waist to hip ratio; HR, heart rate; BP Sys, systolic blood pressure; BP Dia, diastolic blood pressure; HDL, high-density lipoprotein cholesterol; LDL, low-density lipoprotein cholesterol; AST, aspartate aminotransferase; ALT, alanine aminotransferase; GGT, gamma-glutamyl transferase. * At t1 values for AST, ALT and GGT were only measured in 21 participants of the placebo group and values for blood glucose levels were only measured in 21 participants in verum group.

**Table 3:** Fatty acid composition in erythrocytes at baseline and end of study—comparison between verum and placebo groups at baseline (t0) and at 8 weeks (t8) respectively; (Mean values ± standard deviation in %).

% of Total Fatty Acid	Placebo (*n* = 22) t0	Verum (*n* = 22) t0	*p*	Placebo (*n* = 22) t8	Verum (*n* = 22) t8	*p*
Omega-3 Index	4.32 ± 0.35	4.18 ± 0.54	0.318	4.50 ± 0.51	5.72 ± 0.66	<0.001
Omega-3 fatty acids						
C22:6ω3	3.71 ± 0.40	3.64 ± 0.45	0.609	3.87 ± 0.45	4.75 ± 0.60	<0.001
C20:5ω3	0.61 ± 0.16	0.54 ± 0.18	0.175	0.63 ± 0.19	0.97 ± 0.27	<0.001
C18:3ω3	0.14 ± 0.04	0.17 ± 0.06	0.056	0.18 ± 0.08	0.15 ± 0.05	0.170
C22:5ω3	2.41 ± 0.47	2.43 ± 0.42	0.868	2.69 ± 0.44	2.69 ± 0.43	0.995
Omega-6 fatty acids						
C20:4ω6	15.64 ± 1.59	15.40 ± 1.57	0.625	16.49 ± 1.35	15.54 ± 1.55	0.036
C18:2ω6	11.14 ± 1.98	10.86 ± 1.36	0.579	11.96 ± 1.46	11.33 ± 1.68	0.187
C18:3ω6	0.09 ± 0.06	0.08 ± 0.04	0.745	0.09 ± 0.03	0.07 ± 0.03	0.046
C20:2ω6	0.23 ± 0.05	0.24 ± 0.04	0.397	0.22 ± 0.04	0.21 ± 0.04	0.261
C20:3ω6	1.88 ± 0.41	1.93 ± 0.50	0.712	1.97 ± 0.36	1.82 ± 0.44	0.240
C22:4ω6	3.54 ± 0.60	3.52 ± 0.62	0.931	3.49 ± 0.68	3.14 ± 0.73	0.113
C22:5ω6	0.77 ± 0.19	0.80 ± 0.16	0.510	0.73 ± 0.16	0.64 ± 0.23	0.165
Saturated fatty acids						
C14:0	0.46 ± 0.33	0.38 ± 0.09	0.299	0.30 ± 0.10	0.33 ± 0.11	0.268
C16:0	21.67 ± 0.96	21.83 ± 0.80	0.555	21.66 ± 1.11	22.44 ± 1.16	0.029
C18:0	18.39 ± 3.77	18.40 ± 3.81	0.987	16.27 ± 1.13	16.69 ± 2.82	0.526
C20:0	0.14 ± 0.05	0.14 ± 0.04	0.947	0.13 ± 0.03	0.13 ± 0.03	0.504
C22:0	0.26 ± 0.10	0.25 ± 0.09	0.708	0.40 ± 0.11	0.36 ± 0.10	0.187
C24:0	0.89 ± 0.46	0.73 ± 0.43	0.242	0.64 ± 0.28	0.64 ± 0.33	0.938
Cis-monounsaturated fatty acids						
C16:1ω7	0.37 ± 0.16	0.58 ± 0.74	0.195	0.37 ± 0.17	0.41 ± 0.15	0.447
C18:1ω9	15.41 ± 1.28	15.95 ± 1.47	0.200	16.06± 0.65	15.92 ± 1.13	0.628
C20:1ω9	0.28 ± 0.06	0.28 ± 0.04	0.903	0.30 ± 0.06	0.28 ± 0.05	0.070
C24:1ω9	0.92±0.29	0.80 ± 0.29	0.192	0.77 ± 0.17	0.78 ± 0.25	0.910
Trans fatty acids						
C18:2ω6tt	0.16 ± 0.13	0.19 ± 0.13	0.454	0.06 ± 0.05	0.07 ± 0.06	0.695
C18:2ω6ct	0.05 ± 0.03	0.05± 0.03	0.722	0.03 ± 0.04	0.02 ± 0.01	0.578
C18:2ω6tc	0.09 ± 0.03	0.09 ± 0.04	0.969	0.06 ± 0.03	0.06 ± 0.03	0.889
C18:1ω9t	0.47 ± 0.24	0.60 ± 0.31	0.152	0.47 ± 0.15	0.45 ± 0.14	0.542
C16:1ω7t	0.11 ± 0.03	0.11 ± 0.03	0.702	0.15 ± 0.05	0.13 ± 0.04	0.136

Notes: t0, time point for screening visit; t8, time point for end of study visit; *p* = *p*-value. Significant differences in the comparison of baseline to end of study fatty acid compositions in placebo and verum group.

**Table 4:** Comparison of bioavailability of EPA and DHA given in different chemical forms and for different durations.

Chemical Form	Baseline Level EPA + DHA (%)	∆EPA + DHA (%)	EPA + DHA (mg/Day)	∆EPA + DHA (%) /100 mg EPA + DHA	Duration	*n*	Reference
EE	3.30 *	0.87	3320	0.03	2 weeks	9	[23]
	4.18 ± 0.54	1.54	Approximately 250	0.62	8 weeks	22	triOMEG-trial
	5.1 *	3.5	3320	0.11	8 weeks	24	[24]
	4.46 ± 1.13	2.03	907	0.22	8 weeks	26	[25]
	4.46 ± 1.13	4.33	3630	0.12	8 weeks	26	
	6.50 ± 1.46	3.80	3084	0.12	8 weeks	40	[26]
	3.50 *	3.20	2500	0.13	60 days	44	[27]
	4.6 ± 1.5	3.00	1680	0.18	10 weeks	62	[28]
	7.9 ± 3.7	3.4	2520	0.13	12 weeks	11	[29]
	8.27 ± 0.77	2.51	1740	0.14	12 weeks	12	[30]
	3.91 ± 0.30	0.20	440	0.05	12 weeks	15	[31]
	3.54 ± 0.26	1.69	1300	0.13	12 weeks	18	
	3.81 ± 0.23	2.94	2700	0.11	12 weeks	16	
	4.13 *	3.69	720	0.51	16 weeks	43	[32]
	3.7 *	5.4	3360	0.16	16 weeks	30	[33]
	3.74 *	7.24	3360	0.22	16 weeks	122	[34]
	#	4.84	1680	0.29	6 months	39	[35]
	7.6 ± 1.8	4.83	1680	0.29	6 months	39	
	7.4 ± 1.76	4.82	1680	0.29	6 months	45	[36]
FO	5.2 ± 1.7	4.0	4400	0.09	4 weeks	40	[37]
	4.92 *	2.8	1200	0.23	8 weeks	20	[38]
	6.77 *	6.3	3600	0.18	8 weeks	20	
	4.04 *	4.14	3360	0.12	8 weeks	21	[39]
	4.35 *	3.58	3360	0.11	8 weeks	21	
	4.6 ± 1.6	2.9	1680	0.17	10 weeks	36	[40]
	5.74 ± 1.37	5.17	2700	0.19	12 weeks	12	[41]
	9.16 ± 1.74	5.08	2520	0.20	12 weeks	7	[42]
	5.24 ± 0.7	5.46	2700	0.20	12 weeks	10	[43]
	4.90 ± 1.8	4.94	2700	0.18	12 weeks	10	
	5.32 ± 0.74	5.45	2700	0.20	12 weeks	9	[44]
	4.87 ± 1.83	5.05	2700	0.19	12 weeks	7	
	4.33 *	1.83	485	0.38	16 weeks	12	[45]
	4.7 ± 0.9	3.2	500	0.64	5 months	22	[46]
	4.29 ± 0.22	1.90	300	0.63	5 months	23	[47]
	4.28 ± 0.23	2.54	600	0.42	5 months	21	
	4.31 ± 0.22	3.22	900	0.36	5 months	24	
	4.28 ± 0.22	5.21	1800	0.29	5 months	24	
	5.5 *	4.46	3360	0.13	6 months	180	[48]
	4.7 ± 1.1	4.3	1000	0.43	6 months	20	[49]
	3.74 ± 2.02	8.39	2520	0.33	6 months	25	[50]
KO	3.66 ± 0.90	0.31	100	0.31	12 weeks	53	[51]
	3.56 ± 0.82	0.63	200	0.32	12 weeks	53	
	4.00 ± 0.88	1.17	400	0.29	12 weeks	51	
	3.65 ± 0.70	2.65	800	0.33	12 weeks	58	
MO	6.5 *	3.7	1600	0.23	16 weeks	160	[52]
TG	4.37 ± 0.51	2.43	500	0.49	8 weeks	40	[11]
rTG	5.24 *	5.45	2700	0.20	12 weeks	10	[53]
	4.87 *	5.22	2700	0.19	12 weeks	10	
	7.11 ± 1.91	6.20	1680	0.37	6 months	34	[54]
	7.0 ± 1.9	6.25	1680	0.37	6 months	41	[36]
LEM	3.15 *	0.95	657	0.14	2 weeks	10	[23]
	3.34 *	1.22	1314	0.09	2 weeks	8	
	3.14 *	2.18	2628	0.08	2 weeks	10	
AG	6.80 ± 1.93	4.30	2697	0.16	8 weeks	39	[26]
Fish	4.02 *	2.15	485	0.44	16 weeks	11	[45]

In all trials listed, samples were analysed with the method used in the present trial (see Methods). Abbreviations: EE = ethyl-ester, FO = fish oil, KO = krill oil, MO = microalgae oil, TG = triacylglycerides, rTG = re-esterfied triacylglycerides, LEM = liquid emulsion formulation, AG = acylglycerol; * no SD available; # no baseline EPA + DHA level available.
